# Conformational study of quaternary epitope region of V1/V2 loop: influence of disulfide bonds and glycosylations

**DOI:** 10.1186/1742-4690-9-S2-P88

**Published:** 2012-09-13

**Authors:** G Gnanakaran, J Tian, A Sethi

**Affiliations:** 1 Los Alamos National Labs, Los Alamos, USA

## Background

The HIV-1 envelope spike, which consists of a compact, heterodimeric trimer of the glycoprotein gp120 and gp41, is the sole viral target of neutralizing antibodies. The gp120 component of the viral spike is known to be heavily glycosylated, and glycosylation can affect the conformation of envelope spikes. V1/V2 variable loops of gp120 are key target regions for a number of broadly neutralizing human antibodies, such as PG9 and PG16, CH01-CH04, and PGT141-145. Two glycosylation sites (N156 and N160) have been shown by mutagenesis studies to be important in forming the PG9 and PG16 epitopes. Recently, Peter Kwong and coworkers have resolved crystal structure of V1/V2 domain of HIV-1 gp120 from strains CAP45 and ZM109 complexes with antigen-binding fragment of PG9.

## Methods

We employ enhanced molecular dynamics sampling methods (eg. replica exchange molecular dynamics) to dissect the influence of disulfide bond and glycosylation on the conformational landscape of an indel free epitope region of V1/V2 loop. These methods are expected to capture the influence of glycosylation, solvent, rest of the gp120 protein and scaffold constructs on the conformation of V1/V2.

## Results

We evaluate the backbone conformational preferences and solvent accessibility of each residue in the selected V1/V2 region and compare them to the antibody-bound conformation of this region. Both the disulfide bond that links V1 and V2 loops and the nearby glycosylations affect the beta sheet formation propensity of that region. Further characterization indicates that glycans predominantly influence the entropy of the V1/V2 loops.

## Conclusion

Our studies reveal how glycans can impact the electrostatic and hydrophobic surfaces of the V1/V2 regions that have been proposed to form the epitope for broadly neutralizing antibodies. Along with proximal disulfide bonds, glycans tend to affect the local beta-sheet propensities that can further contribute to the quaternary nature of the epitope.

